# Marine Cyanobacteria Compounds with Anticancer Properties: A Review on the Implication of Apoptosis

**DOI:** 10.3390/md10102181

**Published:** 2012-09-28

**Authors:** Margarida Costa, João Costa-Rodrigues, Maria Helena Fernandes, Piedade Barros, Vitor Vasconcelos, Rosário Martins

**Affiliations:** 1 Marine and Environmental Research Center—CIIMAR/CIMAR, Porto University, Rua dos Bragas, 289, 4050-123 Porto, Portugal; Email: costa.anamarg@gmail.com (M.C.); vmvascon@fc.up.pt (V.V.); 2 Laboratory of Pharmacology and Cellular Biocompatibility, Faculty of Dental Medicine, Porto University, Rua Dr. Manuel Pereira da Silva, 4200-393 Porto, Portugal; Email: jrodrigues@fmd.up.pt (J.C.-R.); mhfernandes@fmd.up.pt (M.H.F.); 3 Centre of Health and Environmental Research—CISA, Superior School of Health Technology of Porto, Polytechnic Institute of Porto, Rua Valente Perfeito, 322, 4400-330 Vila Nova de Gaia, Portugal; Email: pgb@estsp.ipp.pt; 4 Faculty of Sciences, Porto University, Rua do Campo Alegre, 4169-007 Porto, Portugal; 5 Institute for Molecular and Cell Biology—IBMC, Porto University, Rua do Campo Alegre 823, 4150-180 Porto, Portugal

**Keywords:** cancer, apoptosis, marine cyanobacteria, natural compounds

## Abstract

Marine cyanobacteria have been considered a rich source of secondary metabolites with potential biotechnological applications, namely in the pharmacological field. Chemically diverse compounds were found to induce cytoxicity, anti-inflammatory and antibacterial activities. The potential of marine cyanobacteria as anticancer agents has however been the most explored and, besides cytotoxicity in tumor cell lines, several compounds have emerged as templates for the development of new anticancer drugs. The mechanisms implicated in the cytotoxicity of marine cyanobacteria compounds in tumor cell lines are still largely overlooked but several studies point to an implication in apoptosis. This association has been related to several apoptotic indicators such as cell cycle arrest, mitochondrial dysfunctions and oxidative damage, alterations in caspase cascade, alterations in specific proteins levels and alterations in the membrane sodium dynamics. In the present paper a compilation of the described marine cyanobacterial compounds with potential anticancer properties is presented and a review on the implication of apoptosis as the mechanism of cell death is discussed.

## 1. Introduction

Cyanobacteria are a diverse group of prokaryotic organisms that can exist in a wide range of ecosystems. Capable to develop photosynthesis, cyanobacteria constitute one of the components of the primary first level organisms in water food chains. These organisms have also important roles in nutrient cycles such as nitrogen cycle, by converting atmospheric nitrogen into an organic form, in a process that releases some residual hydrogen [1].

The first studies concerning cyanobacteria were focused on their ecological and public heath impact, due to their capacity to produce toxins with deleterious effects on plants, invertebrates and vertebrates, including humans [2,3]. In humans, toxins such as microcystins, nodularins and cylindrospermopsin were found to induce liver and kidney damage, cytotoxicity, neurotoxicity, dermal toxicity, gastrointestinal disturbances among others [4]. More recently, several studies have demonstrated that cyanobacteria also produce compounds with biotechnological and pharmaceutical interest. Important biological properties such as anticancer, anti-inflammatory and antibiotic activities have been described [5].

Marine cyanobacteria in particular have been considered a prominent source of structurally diverse and biologically active natural products [6]. The diversity in secondary metabolites is a result of the cyanobacterial capacity to integrate both Non-Ribosomal Peptide Synthethases with Polyketide Synthases. Cyanobacteria have a wide range of enzymes responsible for methylations, oxidations, tailoring and other alterations [7], resulting in chemically diverse natural products such as linear peptides [8], cyclic peptides [9], linear lipopeptides [10], depsipeptides [11], cyclic depsipeptides [12], fatty acid amides [13], swinholides [14], glicomacrolides [15] or macrolactones [16].

A large diversity of biological interactions is described between marine cyanobacteria compounds and several groups of organisms, such as bacteria [17], fungi [18,19] parasites [20] and invertebrates [21]. The role of the compounds in marine environment has been rarely elucidated but a possible explanation is that they represent a defensive handling to the surrounding predators [22]. In what concerns to humans, anti-inflammatory [23] neurotoxic [12] and anticancerigenous [24] are common bioactive properties. The cytotoxic effects of marine cyanobacteria compounds on human tumor cell lines are the most studied, with some compounds producing effects at the nanomolar range [25]. As examples, apratoxin D, produced by species of *Lyngbya* is potently cytotoxic to human lung cancer cells [26] and likewise, symplocamide A, isolated from *Symploca *sp. showed also potent cytotoxicity to lung cancer cells and neuroblastoma cells [27]. 

Cell death is crucial in cancer therapy. Comparing cell death mechanisms in neoplastic cells, apoptosis reveals its importance when compared with necrosis since it occurs as a physiological process to any mild cell injury or simply when cell function is finished or disturbed, occurs via a predictable and coordinated pathway, and cellular deletion does not involve inflammation [28]. In contrast, necrosis is difficult to prevent and always develops an inflammatory response and death of the surrounding cells [29]. Autophagy, also described as a mechanism of cell death, is likewise indicated as a cancer therapeutic target. However, it has a dual effect since maintaining cell survival can promote the growth of established tumors [30]. Several anticancer drugs work as apoptotic modulators, in order to eliminate silent and cleanly the unwanted cells [31,32]. Marine cyanobacteria were found to produce a wide range of compounds that revealed apoptotic properties. Apoptosis can be induced by both intrinsic and extrinsic signals, by multiple agents, as the natural flavonoid quercetin [33], the representative reactive oxygen species H_2_O_2_ [34] or even the UV radiation [35]. Apoptotic cells develop typical morphological alterations that allow its identification. During an early stage of apoptosis, called cell shrinkage, cells have a smaller size, showing a dense cytoplasm with thinner organelles [36]. Martins and co-workers demonstrated that HL-60 cells exposed to aqueous extracts of *Synechocystis *sp. and *Synechococcus *sp. strains, presented cell shrinkage showing that cells were developing apoptosis, and membrane budding, that occurs when cell is fragmented into apoptotic bodies [37]. Apoptotic cells also develop nuclear alterations, visible as nuclear fragmentation and chromatin condensation [36]. Biselyngbyaside, a macrolide glycoside produced by *Lyngbya *sp., was found to induce apoptosis in mature osteoclasts, revealed by nuclear condensation [38]. Marine benthic *Anabaena* sp. extracts were found to induce apoptosis in acute myeloid leukemia cell line, with cells showing several described typical morphological markers, such as chromatin condensation, nuclear fragmentation, surface budding and release of apoptotic bodies [39]. 

Besides morphological markers that allow the direct identification of an apoptotic cell, some other cellular and molecular alterations associated to apoptosis can be identified. Since several marine cyanobacteria compounds interact with important molecular targets involved in anticancer activity leading to a controlled death of tumor cells, this review aims to resume the marine cyanobacterial products that were found to inhibit the proliferation of cancer cell lines, namely by inducing apoptotic cell death. Effects of compounds on cell cycle arrest, mitochondrial dysfunctions and oxidative damage, alterations in caspase cascade, non-caspases proteases involvement, alterations in the Bcl-2 protein family and alterations in membrane sodium channel dynamics are reviewed. In order to summarize the data available in the literature, in Table 1 we present the described cyanobacterial compounds that were found to induce cytotoxic effects on a wide range of cancer cell line, and in Table 2 we describe the most relevant effects related to anticancer activity induced by marine cyanobacteria compounds.

**Table 1:** Marine cyanobacteria compounds with potential anticancer properties.

Compound	Source	Class of compound	Cytoxicity assay	Human cell line tested	Reference
Ankaraholide A	*Geitlerinema*	Glycosilated swinholide	MTT	NCI-H460 lung tumor	[14]
			SRB	MDA-MB-435 breast carcinoma	[14]
Apratoxin A	*Lyngbya majuscula*	Cyclic depsipeptide	SRB	KB oral epidermoid cancer and LoVo colon cancer	[40,41]
			MTT	U2OS osteosarcoma, HT29 colon adenocarcinoma and HeLa cervical carcinoma	[42]
Apratoxins B-C	*Lyngbya *sp.	Cyclic depsipeptides	MTT	KB oral epidermoid cancer and LoVo colon cancer	[40]
Apratoxin D	*Lyngbya majuscula *and *Lyngbya sordida*	Cyclic depsipeptide	MTT	H-460 lung cancer	[26]
Apratoxin E	*Lyngbya bouilloni*	Cyclic depsipeptide	MTT	U2OS osteosarcoma, HT29 colon adenocarcinoma and HeLa epithelial carcinoma	[42]
Apratoxins F and G	*Lyngbya bouilloni*	Cyclic depsipeptides	MTT	H-460 lung cancer	[43]
			Hemocytometer counting	HCT-116 colorectal cancer cells	[43]
Aurilide B	*Lyngbya majuscula*	Cyclic depsipeptide	MTT	H-460 lung tumor	[24]
Aurilide C	*Lyngbya majuscula*	Cyclic depsipeptide	MTT	NCI-H460 lung tumor	[24]
Belamide A	*Symploca *sp.	Linear tetrapeptide	Non-specified	HCT-116 colon cancer	[8]
Bisebromoamide	*Lyngbya *sp.	Peptide	SRB	HeLa S_3 _epithelial carcinoma	[44]
Biselyngbyaside	*Lyngbya *sp.	Glicomacrolide	SRB	HeLa S3 epithelial carcinoma, SNB-78 central nervous system cancer and NCI H522 lung cancer	[15]
Calothrixin A	*Calothrix*	Pentacyclic indolophenanthridine	^3^H-thymidine incorporation	HeLa epithelial carcinoma	[45]
			MTT	Leukemia CEM	[46]
Calothrixin B	*Calothrix*	Pentacyclic indolophenanthridine	MTT	HeLa epithelial carcinoma	[47]
				Leukemia CEM	[46]
Caylobolide A	*Lyngbya majuscula*	Macrolactone	Non-specified	HCT-116 colon tumor	[48]
Caylobolide B	*Phormidium *spp*.*	Macrolactone	MTT	HT29 colorectal adenocarcinoma and HeLa cervical carcinoma	[16]
Coibamide A	*Leptolyngbya *sp.	Cyclic depsipeptide	MTT	Lung cancer NCI-H460, breast cancer MDA-MB-231, melanoma LOX IMVI, leukemia HL-60 and astrocytoma SNB75	[49]
Cryptophycin 1	*Nostoc *spp*.*	Cyclic depsipeptide	Cell morphology examination	MDA-MB-435 mammary adenocarcinoma and SKOV3 ovarian carcinoma	[50]
			AlamarBlue dye reduction	Leukemia U937, CCRF-CEM and HL-60, colon carcinoma HT-29, GC3 and Caco-2, mammary carcinoma MCF-7 and MDA-MB-231 and cervical carcinoma HeLa	[51]
Dolastatin 10	*Symploca *sp.	Linear Pentapeptide	MTT	Lung A549 carcinoma	[52]
				Human lung cancer cells: NCI-H69, -H82, -H446 and -H510	[53]
				Human DU-145 prostate cancer cell line	[54]
			[3H] Thymidine	Several lymphoma cell lines	[55]
			Trypan blue dye	Reh lymphoblastic leukemia	[56]
Dolastatin 12	*Leptolyngbya *sp.	Cyclic depsipeptide	MTT	A549 lung carcinoma	[52]
Dragonamide	*Lyngbya majuscula*	Lipopeptide	Non-specified	A-549 lung epithelial adenocarcinoma, HT-29 colon adenocarcinoma and MEL-28 melanoma	[57]
Ethyl Tumonoate A	*Oscillatoria margaritifera*	Peptide	MTT	H-460 lung cancer	[58]
Hoiamide A	Assemblage of *Lyngbya majuscule and Phormidium *gracile	Cyclic depsipeptide	Non-specified	H-460 lung cancer	[59]
Hoiamide B	Cyanobacterial sample	Cyclic depsipeptide	Non-specified	H-460 lung cancer	[59]
Homodolastatin 16	*Lyngbya majuscula*	Cyclic depsipeptide	MTT	WHCO1 and WHCO6 esophageal cancer and ME180 cervical cancer	[60]
Isomalyngamide A and A-1	*Lyngbya majuscula*	Fatty acid amides	MTT	Breast cancer MCF-7 and MDA-MB-231	[13]
Jamaicamides A-C	*Lyngbya majuscula*	Polyketide-Peptides	MTT	H-460 lung cancer	[61]
Kalkitoxin	*Lyngbya majuscula*	Lipopeptide	Trypan blue dye	HCT-116 colon	[62]
Lagunamide C	*Lyngbya majuscula*	Cyclic depsipeptide	MTT	Lung adenocarcinoma A549, cancer prostate PC3, ileocecal colorectal cancer HCT8 and ovary cancer SK-OV	[63]
Largazole	*Symploca *sp.	Cyclic depsipeptide	MTT	MDA-MB-23I breast cancer and U2OS osteosarcoma	[64]
				A549 lung cancer and HCT-116 colorectal carcinoma	[65]
Lyngbyabellin A	*Lyngbya majuscula*	Cyclic depsipeptide	Non-specified	KB nasopharyngeal carcinoma and LoVo colon adenocarcinoma	[66]
Lyngbyaloside	*Lyngbya *sp.	Glicomacrolide	Non-specified	KB nasopharyngeal carcinoma and LoVo colon adenocarcinoma	[67]
Majusculamide C	*Lyngbya majuscula*	Cyclic depsipeptide	Non-specified	Ovarian carcinoma OVCAR-3, kidney cancer A498, lung cancer NCI-H460, colorectal cancer KM20L2 and glioblastoma SF-295	[68]
Malevamide D	*Symploca hydnoides*	Peptide ester	Non-specified	Lung cancer A-549, colon cancer HT-29 and melanoma MEL-28.	[69]
Malyngamide 2	*Lyngbya sordida*	Fatty acid amine	MTT	H-460 lung cancer	[23]
Malyngamide C, J and K	*Lyngbya majuscula*	Fatty acid amines	MTT	H-460 lung cancer	[70]
Malyngolide dimmer	*Lyngbya majuscula*	Cyclodepside	MTT	H-460 lung cancer	[71]
Nostocyclopeptide A1 and A2	*Nostoc *sp.	Cyclic heptapeptides	Non-specified	KB oral epidermoid cancer and LoVo colon cancer	[72]
Obyanamide	*Lyngbya confervoides*	Cyclic depsipeptide	Non-specified	KB oral epidermoid cancer and LoVo colon cancer	[73]
Palauamide	*Lyngbya *sp.	Cyclic depsipeptide	Non-specified	Cervical carcinoma HeLa, lung adenocarcinoma A549 and gastrocarcinoma BGC	[74]
				KB oral epidermoid cancer	[75]
Palmyramide A	*Lyngbya majuscula*	Cyclic depsipeptide	MTT	H-460 lung cancer	[76]
Pitipeptolides A-B	*Lyngbya majuscula*	Cyclic depsipeptides	Non-specified	LoVo colon cancer	[77]
			MTT	HT29 colon adenocarcinoma and MCF-7 breast cancer	[17]
Pitipeptolide C	*Lyngbya majuscula*	Cyclic depsipeptide	MTT	HT29 colon adenocarcinoma and MCF-7 breast cancer	[17]
Pitiprolamide	*Lyngbya majuscula*	Cyclic depsipeptide	MTT	HCT116 colorectal carcinoma and MCF7 breast adenocarcinoma	[78]
Pseudodysidenin	*Lyngbya majuscula*	Lipopeptide	Non-specified	A-549 lung adenocarcinoma, HT-29 colon adenocarcinoma and MEL-28 melanoma	[57]
Somocystinamide A	*Lyngbya majuscula*	Lipopeptide	XTT	Jurkat and CEM leukemia, A549 lung carcinoma, Molt4 T leukemia, M21 melanoma and U266 myeloma	[79]
Symplocamide	*Symploca *sp.	Cyclic peptide	Non-specified	H-460 lung cancer	[27]
Symplostatin 1	*Symploca hydnoides*	Linear Pentapeptide	SRB	MDA-MB-435 breast carcinoma and NCI/ADR ovarian carcinoma	[25]
				Epidermoid carcinoma cell line	[80]
Tasiamide	*Symploca *sp.	Cyclic peptide	Non-specified	KB oral epidermoid cancer and LoVo colon cancer	[81]
Tasiamide B	*Symploca *sp.	Peptide	Non-specified	KB oral epidermoid cancer	[82]
Tasipeptins A-B	*Symploca *sp.	Cyclic depsipeptides	Non-specified	KB oral epidermoid cancer	[83]
Ulongapeptin	*Lyngbya *sp.	Cyclic depsipeptide	Non-specified	KB oral epidermoid cancer	[84]
Veraguamides A-G	*Symploca cf. hydnoides*	Cyclic depsipeptides	MTT	H-460 lung cancer	[85]
Wewakazole	*Lyngbya sordida*	Cyclic dodecapeptide	MTT	H-460 lung cancer	[23]
Wewakpeptins	*Lyngbya semiplena*	Depsipeptides	MTT	H-460 lung cancer	[11]

MTT: 3-(4,5-dimethylthiazolyl-2)-2,5-diphenyltetrazolium bromide; XTT: 2,3-bis-(2-methoxy-4-nitro-5-sulfophenyl)-2*H*-tetrazolium-5-carboxanilide; SBR: Sulforhodamine B.

**Table 2:** Relevant anticancer cell effects induced by marine cyanobacteria compounds.

Compound	Source	Class of compound	Model tested	Cell effect	Reference
Alotamide	*Lyngbya bouillonii*	Cyclic depsipeptide	Murine cerebrocortical neurons	Calcium inﬂux promotion	[12]
Ankaraholide A	*Geitlerinema*	Glycosilated swinholide	Rat aorta A-10 cells	Loss of filamentous (F)-actin	[14]
Antillatoxin	*Lyngbya majuscula*	Lipopeptide	Primary rat cerebellar granule cells	Voltage-gated sodium channel activation	[86]
			CHL 1610 Chinese hamster lung cells		[87]
Antillatoxin B	*Lyngbya majuscula*	Lipopeptide	neuro-2a mouse neuroblastoma cells	Sodium channel activation	[10]
Apratoxin A	*Lyngbya majuscula*	Cyclic depsipeptide	Human HeLa cervical carcinoma cells	Cell cycle inhibition	[88]
			Human U2OS osteosarcoma cells	Secretory pathway inhibition	[89]
Aurilide B	*Lyngbya majuscula*	Cyclic depsipeptide	Rat aorta A-10 cells	Microfilament disruption	[24]
Belamide A	*Symploca *sp.	Linear tetrapeptide	Rat aorta A-10 cells	Microtubule disruption	[8]
Bisebromoamide	*Lyngbya *sp.	Peptide	Human HeLa epithelial carcinoma cells	Actin filaments stabilization	[90]
			Normal rat kidney cells extracellular signal regulated protein kinase	Protein kinase inhibition	[44]
Bouillomides A-B	*Lyngbya bouillonii*	Depsipeptides	Elastase and chymotrypsin	Serine proteases inhibition	[91]
Calothrixin A	*Calothrix*	Pentacyclic indolophenanthridine	Human leukemia CEM cells	Cell cycle inhibition	[46]
Calothrixin B	*Calothrix*	Pentacyclic indolophenanthridine	Human HeLa epithelial carcinoma cells	Cell cycle inhibition	[45]
			Human HeLa epithelial carcinoma cells	Oxidative stress induction	[45]
Coibamide A	*Leptolyngbya *sp.	Cyclic depsipeptide	Human NCI-H460 lung cancer cell line	Cell cycle inhibition	[49]
Cryptophycin 1	*Nostoc *spp*.*	Cyclic depsipeptide	Human MDA-MB-435 mammary adenocarcinoma and SKOV3 ovarian carcinoma cells	Cell cycle inhibition	[50]
			Human MDA-MB-435 mammary adenocarcinoma	Caspase-3 protein activation	[50]
Curacin A	*Lyngbya majuscula*	Lipopeptide	Tubulin	Tubulin polymerization inhibition	[92]
			Human A549 lung carcinoma cells	Bad protein levels increase	[52]
			Human A549 lung carcinoma cells	Caspase-3 protein activation	[52]
			Bovine β-tubulin	Tubulin polymerization inhibition	[93]
Dolastatin 10	*Symploca *sp.	Linear Pentapeptide	Human Reh lymphoblastic leukemia cells	Bcl-2 protein reduction	[56]
			Human lung cancer cells: NCI-H69 and -H510	Bcl-2 protein phosphorylation	[53]
			Human A549 lung carcinoma cells	Bad protein levels increase	[52]
			Human A549 lung carcinoma cells	Caspase-3 protein activation	[52]
Dolastatin 12	*Leptolyngbya *sp.	Cyclic depsipeptide	Rat aorta A-10 cells	Microfilament disruptor	[94]
Grassystatin A-B	*Lyngbya confervoides*	Linear depsipeptides	Cathepsins D and E	Proteases inhibition	[95]
Hectochlorin	*Lyngbya majuscula*	Lipopeptide	Human CA46 Burkitt lymphoma cells	Cell cycle inhibition	[18]
Hermitamides A-B	*Lyngbya majuscula*	Lipopeptide	Human HEK embryonic kidney cells	Voltage-gated sodium channel inhibition	[96]
Hoiamide A	Assemblage of *Lyngbya majuscule *and *Phormidium gracile*	Cyclic depsipeptide	Primary cultures of neocortical neurons from embryonic mice	Sodium channel activation	[59,97]
Hoiamide B	Cyanobacterial sample	Cyclic depsipeptide	Primary cultures of neocortical neurons from embryonic mice	Sodium influx stimulation	[59]
Kalkitoxin	*Lyngbya majuscula*	Lipopeptide	Primary rat cerebellar granule neuron cultures	Calcium influx inhibition	[98]
Kempopeptin A	*Lyngbya *sp.	Cyclic depsipeptide	Bovine pancreatic α-chymotrypsin, porcine pancreatic elastase	Serine Protease Inhibition	[99]
Kempopeptin B	*Lyngbya *sp.	Cyclic depsipeptide	Trypsin	Serine Protease Inhibition	[99]
Largamides A-C	*Lyngbya confervoides*	Cyclic depsipeptides	Porcine pancreatic elastase	Serine protease inhibition	[100]
Largamides D-G	*Oscillatoria *sp.	Cyclic depsipeptides	α-chymotrypsin	Serine protease inhibition	[101]
Lyngbyabellin A	*Lyngbya majuscula*	Cyclic depsipeptide	Human CA46 Burkitt lymphoma cells	Cell cycle inhibition	[18]
			Rat aorta A-10 cells	Microfilament disruption	[66]
Lyngbyabellin B	*Lyngbya majuscula*	Cyclic depsipeptide	Human CA46 Burkitt lymphoma cells	Cell cycle inhibition	[18]
Lyngbyastatin 1	*Lyngbya majuscula*	Cyclic depsipeptide	Rat aorta A-10 cells	Microfilament disruption	[94]
Lyngbyastatin 4	*Lyngbya confervoides*	Cyclic depsipeptide	Bovine pancreatic α-chymotrypsin and porcine pancreatic elastase	Serine protease inhibition	[102]
Lyngbyastatin 5-7	*Lyngbya *spp*.*	Cyclic depsipeptides	Porcine pancreatic elastase	Serine protease inhibition	[103]
Lyngbyastatin 8-10	*Lyngbya semiplena*	Cyclic depsipeptides	Porcine pancreatic elastase	Serine protease inhibition	[104]
Malevamide E	*Symploca laete-viridis*	Depsipeptide	Human HEK embryonic kidney cells	Calcium influx inhibition	[105]
Molassamide	*Dichothrix utahensis*	Depsipeptide	Bovine pancreatic α-chymotrypsin and porcine pancreatic elastase	Serine protease inhibition	[106]
Palmyramide A	*Lyngbya majuscula*	Cyclic depsipeptide	Mouse neuroblastoma neuro-2a cells	Sodium channel inhibition	[76]
Palmyrolide	Assemblage of *Leptolyngbya *cf. and *Oscillatoria *spp*.*	Macrolide	Mouse neuroblastoma neuro-2a cells	Sodium influx inhibition	[107]
			Murine cerebrocortical neurons	Inhibition of calcium oscillations	[107]
Pitipeptolides A and B	*Lyngbya majuscula*	Cyclic depsipeptides	Elastase	Serine protease stimulation	[77]
Pompanopeptin A	*Lyngbya confervoides*	Cyclic peptide	Porcine pancreatic trypsin	Serine protease inhibition	[108]
Symplocamide	*Symploca *sp.	Cyclic peptide	Chymotrypsin	Serine protease inhibition	[27]
Symplostatin 1	*Symploca hydnoides*	Linear Pentapeptide	Rat aorta A-10 and human HeLa cervical carcinoma cells	Cell cycle inhibition	[25]
			Rat aorta A-10 cells	Microtubule depolymerization	[109]
			Human MDA-MB-435 breast carcinoma cells	Bcl-2 phosphorylation	[25]
			Human MDA-MB-435 breast carcinoma cells	Caspase-3 protein activity stimulation	[25]
Symplostatin 3	*Symploca *sp.		Rat aorta A-10 cells	Microtubule depolymerization	[110]
Tiglicamides A-C	*Lyngbya confervoides*	Cyclic depsipeptides	Porcine pancreatic elastase	Serine protease inhibition	[111]

## 2. Cell Cycle Arrest

**Figure 1:**
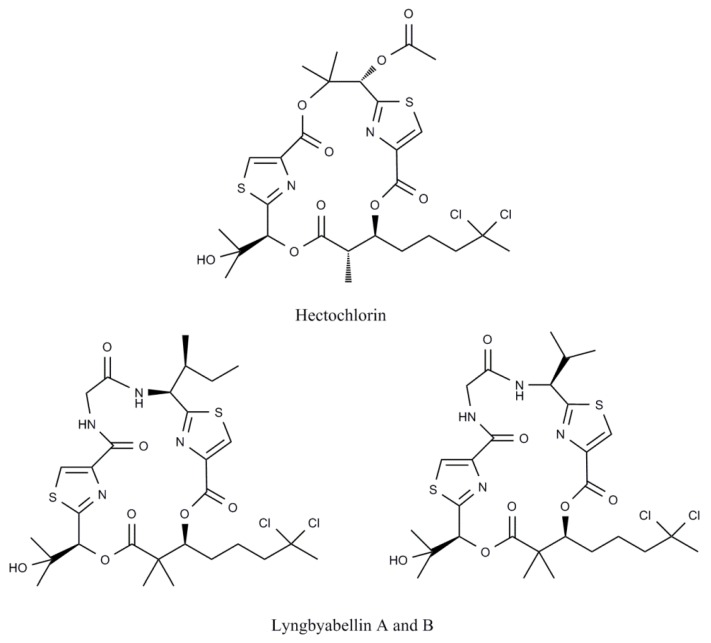
Chemical structures of the marine cyanobacterial secondary metabolites hectochlorin and lyngbyabellins A and B.

Cell cycle is a delicate mechanism that comprises cell growth and its division into two daughter cells. Some substances are able to disturb the normal functioning of this mechanism compromising cell viability, a consequence that can be directly related with apoptosis. A common cellular damage induced by marine cyanobacteria compounds is the disruption of microtubules and actin proteins [112]. As these proteins are directly involved in mitosis, alterations in the normal functioning of the cell cycle occur. The most frequent consequence is G_2_/M phase arrest. Cryptophycin 52, a macrocyclic depsipeptide analogue of the naturally occurring cryptophycins isolated from the marine cyanobacteria *Nostoc* spp. [113], and calothrixin A, a indolophenanthridine isolated from *Calothrix*, are two examples of bioactive metabolites that induced, in different human cancer cell lines, a cell cycle arrest in G_2_/M phase [45]. Dolastatins are cytotoxic peptides that were initially isolated from the sea hare *Dolabella auricularia* and later found to be produced by marine cyanobacterial strains [109]. To explore their anticancer potential, several synthetic analogues were produced. Dolastatin 10, found in *Symploca*, and its non-cyanobacterial analogue, dolastatin 15, were both found to induce an arrest in the same cell cycle phase, G_2_/M phase, inducing apoptosis [52,114]. Symplostatin 1, another analogue of dolastatin 10 and cryptophycin 1, a dolastatin 52 analogue, were also responsible for a G_2_/M arrest in human cancer cells and for disturbances in the formation of mitotic spindles [25,113,114]. Calothrixin A, beyond an arrest in G_2_/M phase in a leukemia cell line at 1 μM and 10 μM, is also responsible for a cumulative arrest in S phase [46]. Hectochlorin and lyngbyabellins are structurally related lipopeptide and cyclic depsipeptides isolated from the genus *Lyngbya*. Both hectochlorin and lyngbyabellin B are described to induce an arrest in G_2_/M phase in a human Burkitt lymphoma cell line, accompanied with a related increase in binucleated cells and an apparent thickening of the microfilaments [18]. Nagarajan and co-workers [115] suggested that the inhibition of cell cycle proliferation by lyngbyabellins is assigned to a thiazole ring and dichlorinated components (Figure 1), once these compounds were all found to inhibit cell cycle proliferation [18,116]. 

Besides G2/M phase arrest effects in G1 phase are also described. Khan and co-workers [46] reported a G_1_ phase arrest after treatment with a low concentration (0.1 μM) of calothrixin B. The same effect was demonstrated by Ma *et al*. [88] in a cervical carcinoma cell line treated with the cyclic depsipeptide apratoxin A (50 nM). Coibamide, a potent cytotoxic cyclic depsipeptide, founded in a Panamanian *Leptolyngbya *sp., was also described as capable to cause a significant dose dependent increase in the number of cells in G_1_ phase of the cell cycle [49].

## 3. Mitochondrial Dysfunctions and Oxidative Damage

Mitochondria have essential functions in aerobic cells, and interferences in its normal behavior are crucial to determine cell fate [117]. A dysfunction in these organelles imbalances the cell redox potential, inducing damages in cell components that can lead, in the cases that pro-survival mechanisms fail, to apoptosis [118]. To the best of our knowledge, no study relating marine cyanobacterial natural products with mitochondrial dysfunctions has been done. However aurilide, a cyclodepsipeptide isolated from the sea hare *Dolabella auricularia* and related with the marine cyanobacterial aurilides A and B, is described to induce a dysfunction in mitochondria. HeLa cells, when treated with this metabolite exhibited mitochondria fragmentation, visible by MitoTracker Red staining [119]. 

Oxidative stress is a cell condition that can be triggered by mitochondrial disorders. It can occur due to an overproduction of reactive oxygen species (ROS) or to a decrease in antioxidant levels [120]. Calothrixin A is described as an oxidative stress inducer in Jurkat human T cells, since they show an increase on intracellular ROS content after treatment with that molecule [45]. DNA damage is also a consequence directly associated to the oxidative stress, and it is commonly observed as a result of exposure to cyanobacterial secondary metabolites. As expected, besides an increase in ROS, calothrixin A foments DNA fragmentation [45]. DNA fragmentation is the most common DNA damage observed. Dolastatin 10 induced DNA damage on several human lymphoma cell lines [55] and on lung cancer cells [52]. Cryptophycins 1 and 52 are also metabolites that were found to induce DNA fragmentation [50,113]. 

External nuclei alterations can be also a consequence of oxidative stress. Binucleated cells are frequently observed as a response to cyanobacterial products, as swinholide A, isolated from cyanobacterial samples of *Symploca *cf. sp. [14] or lyngbyabellin [116]. Symplostatin 1 was found to induce an abnormal nuclear convolution in a rat aorta cell line, leading to the breakdown of nucleus and the formation of numerous micronuclei [25].

To counterbalance the deleterious effects of ROS, cells developed a complex antioxidant system. The antioxidant enzymes, like superoxide dismutase (SOD), catalase, glutathione-*S*-transferase (GST) and several peroxidases, constitute the front line, with important scavenging functions. Some other molecules, with low molecular weight, have crucial roles, such as glutathione, ascorbate or phenolic compounds [121]. The capacity of marine cyanobacterial natural products to interfere with the antioxidant system of human cells is not well elucidated. Evidences indicate that pigments are the compounds with higher antioxidant activity. Carotenoids isolated from the marine *Trichodesmium* are responsible for an antioxidative protection, observed with ferric reducing/antioxidant power assay [122]. In the same study, extracts from marine strains of *Anabaena*, *Cyanothece*, *Prochlorothrix *and *Synechococcus* showed antioxidant properties, but mainly in the protein extract [122]. Also the major phycobiliprotein, c-phycocyanin, from both *Lyngbya* and *Phormidium*, is capable to scavenge ROS, in particular peroxyl and hydroxyl radicals [123]. It was also suggested that this antioxidant capacity is resultant from the covalent linked tetrapyrole chromophore with phycocyanobilin [123]. 

## 4. Alterations in Caspase Cascade

Caspases are a family of cysteine aspartate proteases that act as the central executers of apoptosis. They are synthesized as inactive zymogens, which are activated after proteolytic cleavage [124]. According to their point of entrance into apoptotic process, caspases can be classified as initiators or effectors. Initiator caspases, that include -8, -9, and -10, activate the downstream effectors caspases, -3, -6 and -7, in a cascade of events that triggers a controlled and programmed cell death [125].

Marine cyanobacteria produce several compounds that are capable to induce alterations on caspases as a pathway to induce cell death. Several marine benthic cyanobacterial extracts showed to induce apoptosis partially dependent of protein caspases. Cells overexpressing LEDGF/p75, an inhibitor of cell death dependent of caspases, showed an increase in just a few number of apoptotic cells after treatment, when compared with the control [39].

Caspase-3 is the most studied caspase concerning to apoptosis induced by natural products. The activity of caspase-3 protein is increased after exposure to symplostatin 1 [25] and to the glicomacrolide biselyngbyaside [38]. Also cryptophycin 1 is described to induce apoptosis in a human ovarian carcinoma cell line, initiating the caspases cascade through caspase-3 activation [50]. The cleavage, and therefore the activation, of caspase-3 were still previously observed as a response to dolastatins 10 and 15 and to the lipopeptide curacin A [52].

Cryptophycin 52 induced an apoptosis dependent on both caspase-3 and caspase-1 activation [113]. Another study [79] also reported that apoptosis induced by somocystinamide A, a lipopeptide from *Lyngbya majuscula*, occurs in a caspase-8 dependent manner, since it was observed an inhibition of tumor growth selectively in the caspase-8-expressing neuroblastoma cells, when compared with cells lacking the protein.

## 5. Non-Caspases Proteases Involvement

Although caspases have a central role in the apoptotic cell death developing, it is described that the process often continues after an inhibition of this proteins [126,127]. This finding suggests the implication of other executors, which promote apoptosis in the absence of caspases. It was already proposed that some other proteases, capable to support apoptosis, have caspases amplification and assistance functions [128]. 

**Figure 2:**
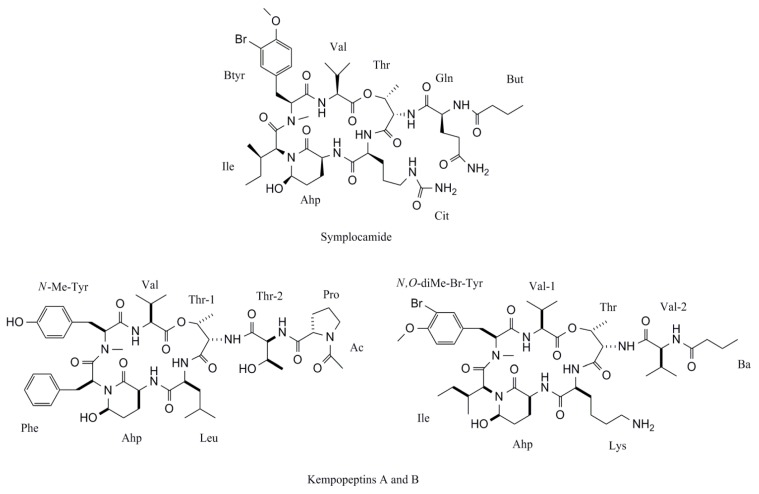
Chemical structures of the marine cyanobacterial secondary metabolites symplocamide and kempopeptins A and B.

Proteases are involved in the irreversibly hydrolysis of the peptide bonds in proteins, an important post-translational modification. These proteolytic enzymes are important for the control of a large number of key physiological processes, including apoptosis [129]. Apoptotic cell death induced by intracellular proteolysis of some serine proteases is already described [130]. Several cyanobacterial compounds have been described to interfere with the normal functioning of serine proteases, mainly the pancreatic elastase, chymotrypsin and trypsin, as is resumed in Table 3. Symplocamide A was described to inhibit chymotrypsin with an IC_50_ of 0.38 μM, with trypsin being also affected but with an IC_50_ of 80.2 μM, a difference greater than 200-fold [27]. The authors suggested that, to inhibit trypsin under 10 μM, a basic aminoacid residue between treonine (Thr) and 3-amino-6-hydroxy-2-piperidone (Ahp) is needed. A hydrophobic and neutral residue in this position confers to the compound a preference for chymotrypsin. Kempopeptins A and B are other two cyclodepsipeptides isolated from a Floridian collection of a marine *Lyngbya *sp. that reveal a strong potency to inhibit proteases activity [99]. Kempopeptin B, with a leucine (Leu) residue between Thr and Ahp (Figure 2), only inhibit trypsin activity (IC_50_ = 8.4 μM), but kempopeptin A, with a lysine (Lys) in the same position, inhibit both elastase (IC_50_ = 0.32 μM) and chymotrypsin (IC_50_ = 2.6 μM). Bouillomides A and B, two depsipeptides isolated from *Lyngbya bouillonii* and molassamide, a depsipeptide from *Dichothrix utahensis*, all dolastatin 13 analogues, contain 2-aminobutyric acid (Abu) between Thr and Ahp. As expected, these metabolites are chymotrypsin inhibitors [91,106]. Largamides are another family of cyclic peptides isolated from *Lyngbya confervoides*. Largamides D and E, with a Leu residue between Thr and Ahp, and largamides F and G, with a tyrosine (Tyr) in the same position, also inhibited chymotrypsin with IC_50_ range from 4.0 to 25.0 μM [101]. 

Pompanopeptin A, a cyclic peptide isolated from the *Lyngbya confervoides* and kempopeptin B, contain arginine (Arg) and lysine (Lys), respectively, between Thr and Ahp. These basic residues give to the compounds the capacity to inhibit trypsin, pompanopeptin with an IC_50_ of 2.4 μM and kempopeptin with 8.4 μM [99,108].

Ahp residue-containing natural products are responsible for the inhibition of elastase [99]. Lyngbyastatins 4–10, a group of compounds that contain the Ahp residue, were all described as elastase inhibitors [102,103,104] with an IC_50_ range from 0.03 (lyngbyastatin 4) to 210 μM (lyngbyastatin 9). Lyngbyastatins are also strong chymotrypsin inhibitors, but with less potency than elastase, IC_50_ = 0.3 μM [99]. The same profile is verified with the depsipeptide molassamide witch contains the Ahp residue, which is capable to inhibit the elastase activity [106]. Largamides A–C and tiglicamides A–C, depsipeptides isolated from *Lyngbya confervoides* are non-containing Ahp natural compounds. However, these products were all responsible for an elastase enzyme inhibition [100,111].

**Table 3:** Marine cyanobacteria natural products with an inhibitory effect in serine proteases.

Compound	Source	Class of compound	Serine protease inhibition			Reference
			Elastase	Chymotripsin	Thrypsin	
Bouillomide A	*Lyngbya bouillonii*	Depsipeptide	IC_50_ = 1.9 μM	IC_50_ = 0.17 μM	No inhibition at 100 μM	[91]
Bouillomide B	*Lyngbya bouillonii*	Depsipeptide	IC_50_ = 1.0 μM	IC_50_ = 9.3 μM	No inhibition at 100 μM	[91]
Kempopeptin A	*Lyngbya *sp.	Cyclic depsipeptide	IC_50_ = 0.32 μM	IC_50_ = 2.6 μM	IC_50_ > 67 μM	[99]
Kempopeptin B	*Lyngbya *sp.	Cyclic depsipeptide	IC_50_ > 67 μM	IC_50_ > 67 μM	IC_50_ = 8.4 μM	[99]
Largamide A	*Lyngbya confervoides*	Cyclic depsipeptide	IC_50_ = 1.41 μM	No inhibition at 50 μM	No inhibition at 50 μM	[100]
Largamide B	*Lyngbya confervoides*	Cyclic depsipeptide	IC_50_ = 0.53 μM	No inhibition at 50 μM	No inhibition at 50 μM	[100]
Largamide C	*Lyngbya confervoides*	Cyclic depsipeptide	IC_50_ = 1.15 μM	No inhibition at 50 μM	No inhibition at 50 μM	[100]
Largamide D	*Oscillatoria *sp.	Cyclic depsipeptide	Not described	IC_50_ = 10.0 μM	No inhibition	[101]
Largamide E	*Oscillatoria *sp.	Cyclic depsipeptide	Not described	IC_50_ = 10.0 μM	No inhibition	[101]
Largamide F	*Oscillatoria *sp.	Cyclic depsipeptide	Not described	IC_50_ = 4.0 μM	No inhibition	[101]
Largamide G	*Oscillatoria *sp.	Cyclic depsipeptide	Not described	IC_50_ = 25.0 μM	No inhibition	[101]
Lyngbyastatin 4	*Lyngbya confervoides*	Cyclic depsipeptide	IC_50_ = 0.03 μM	IC_50_ = 0.30 μM	No inhibition at 30 μM	[102]
Lyngbyastatin 5	*Lyngbya *spp*.*	Cyclic depsipeptide	IC_50_ = 3.2 μM	IC_50_ = 2.8 μM	No inhibition at 30 μM	[103]
Lyngbyastatin 6	*Lyngbya *spp*.*	Cyclic depsipeptide	IC_50_ = 2.0 μM	IC_50_ = 2.5 μM	No inhibition at 30 μM	[103]
Lyngbyastatin 7	*Lyngbya *spp*.*	Cyclic depsipeptide	IC_50_ = 3.3 μMIC_50_ = 0.47 μM	IC_50_ = 2.5 μM	No inhibition at 30 μM	[103,104]
Lyngbyastatin 8	*Lyngbya semiplena*	Cyclic depsipeptide	IC_50_ = 0.12 μM	Not described	Not described	[104]
Lyngbyastatin 9	*Lyngbya semiplena*	Cyclic depsipeptide	IC_50_ = 0.21 μM	Not described	Not described	[104]
Lyngbyastatin 10	*Lyngbya semiplena*	Cyclic depsipeptide	IC_50_ = 0.12 μM	Not described	Not described	[104]
Molassamide	*Dichothrix utahensis*	Depsipeptide	IC_50_ = 0.032 μM	IC_50_ = 0.234 μM	No inhibition at 10 μM	[106]
Pompanopeptin A	*Lyngbya confervoides*	Cyclic peptide	Not described	Not described	IC_50_ = 2.4 μM	[108]
Symplocamide A	*Symploca *sp.	Cyclic peptide	Not described	IC_50_ = 0.38 μM	IC_50_ = 80.2 μM	[27]
Somamide B	*Lyngbya majuscula *and* Schizothrix* assemblage	Depsipeptide	IC_50_ = 9.5 μM	IC_50_ = 4.2 μM	No inhibition at 30 μM	[103]
Tiglicamide A	*Lyngbya confervoides*	Cyclic depsipeptide	IC_50_ = 2.14 μM	Not described	Not described	[111]
Tiglicamide B	*Lyngbya confervoides*	Cyclic depsipeptide	IC_50_ = 6.99 μM	Not described	Not described	[111]
Tiglicamide C	*Lyngbya confervoides*	Cyclic depsipeptide	IC_50_ = 7.28 μM	Not described	Not described	[111]

Pitipeptolides A and B, two cyclodepsipeptides isolated from the marine cyanobacteria *Lyngbya majuscula *collected at Guam, revealed a particular bioactivity. When in contact to elastase, these compounds induce a significant increase in activity: 2.76-fold and 2.55-fold, respectively, at 50 μg/mL [77]. The authors suggested that this biological activity can be attributed to the presence of hydrophobic portions in the molecule [77].

Cathepsin D is a lysosomal protease that was described to have both anti-apoptotic [131] and pro-apoptotic functions [132]. Cathepsin E, besides its function being not well studied, it was described as a cathepsin D-like protein [133]. Grassystatins A and B, two linear depsipeptides isolated from *Lyngbya confervoides* were found to strongly inhibit cathepsins D (IC_50_ = 26.5 nM and 7.27 nM, respectively) and E (IC_50_ = 886 pM and 354 pM) [95].

## 6. Alterations in the Bcl-2 Protein Family

The Bcl-2 protein family is one of the major apoptosis regulators, which functions in the modulation of the outer mitochondrial membrane. The antiapoptotic members Bcl-2 and Bcl-x_L_ protect the membrane integrity and avoid the release of the cytochrome *c*, but their activity can be disturbed by the pro-apoptotic members Bax, Bad and Bid [28].

Symplostatin 1 initiates the phosphorylation of Bcl-2, inhibiting its anti-apoptotic properties in human breast cancer cells and the total content of the protein appear also to be decreased [25]. Exposure to cryptophycin 52 was responsible for Bcl-2 and Bcl-x_L_ phosphorylation in several prostate cancer cell lines [113]. Dolastatin 10 was associated to a Bcl-2 protein content reduction [56] and suggested to induce phosphorylation of the protein [53]. These are the common defensive mechanisms, the anti-apoptotic members are downregulated by phosphorylation, to allow the mechanisms of cell survival. However, cells can develop different ways of protection and, surprisingly, the synthetic analogue of dolastatin 10, dolastatin 15, promotes the overexpression of Bcl-2 protein in four different lung cancer cell lines [134].

Catassi and co-workers studied the response of non-small cell lung cancer cells when treated with curacin and dolastatins 10 and 15 and observed that these compounds inhibit Bad phosphorylation at serine^136^ [52]. The authors propose that the complex allow Bad to move into the mitochondria and promotes cytochrome *c* release, to trigger apoptosis [52]. Apart from the cell mechanism developed, the Bcl-2 protein family seems to play a crucial role in apoptosis induced by marine cyanobacterial natural compounds.

## 7. Alterations in Membrane Sodium Channel Dynamics

In mammal cells, a concentration gradient is necessary to keep the high levels of intracellular potassium and the low levels of sodium. This gradient is held by several ionic transporters and channels and by the capacity of cells to adapt to non-isotonic conditions, by volume regulatory mechanisms [135]. In apoptosis, a disordered volume regulation that leads to cell shrinkage during regular osmotic conditions occurs [136] leading to an early increase in the intracellular sodium concentration [137].

Marine cyanobacterial natural compounds seem to be involved in both induction and inhibition of sodium channels in neural cells. Antillatoxin, a lipopeptide isolated from *Lyngbya majuscule* was responsible for a rapid increase in sodium concentration inside of the cell in primary rat cerebellar granule cells [86]. Although the mechanism of interaction is not well described, the authors excluded an interaction of antillatoxin with channel neurotoxin sites 1–3, 5 and 7. Hoiamides are a class of cyclic depsipeptides with sodium channel bioactivity [59,97]. Hoiamides A and B were described to activate sodium channels in primary cultures of neocortical neurons from embryonic mice, with an IC_50_ of 1.7 μM and 3.9 μM, respectively [59]. In another work it was suggested that hoiamide A acts as a partial agonist at neurotoxin site 2 [97].

Palmyramide A, a cyclic depsipeptide from *Lyngbya majuscule*, showed to inhibit a veratridine and ouabain induced sodium overload with an IC_50_ value of 17.2 μM. The authors suggested that the inhibition may occur by blocking the voltage-gated sodium channel [76]. Palmyrolide, a macrolide isolated from an assemblage of *Leptolyngbya *cf. and *Oscillatoria *spp*.*, is a stronger inhibitor of veratridine and ouabain induced sodium overload with an IC_50_ value of 3.70 μM [107].

Hermitamides A and B are two lipopeptides, isolated from the marine cyanobacteria *Lyngbya majuscula *from a Papua New Guinea collection. Hermitamide A is a sodium channel blocker that inhibits it near to 50% at 1 μM. Hermitamide B is a more potent blocker, inhibiting near to 80% at 1 μM [96]. It was proposed that the aromatic region of these compounds is important for the channel inhibition, being the indole group of hermitamide B an advantage over the phenyl ring of hermitamide A. A bioinformatic approach reveals that the connection between hermitamide B and human voltage-gated sodium channel is driven mainly by a hydrophobic interaction with residue K1237, and H-bonds between the amide group of hermitamide B with N434 and Y1586. Hydrophobic interactions between hermitamide B and F1283, F1579, L1582, V1583, Y1586, L1280, L788, F791, L792, I433, and L437 residues are also predicted [96]. 

Alterations in intracellular sodium levels and the interaction between cyanobacterial natural products and the sodium channels are important keys to understand the toxic mechanism and to develop possible pharmacological applications. 

## 8. Conclusions

Marine cyanobacteria have been identified as one of the most promising groups of organisms from which novel biochemically active natural products, with potential benefits against cancer, can be isolated. Although several compounds were found to inhibit cell growth in a large variety of cancer cell lines, the pathways by which cancer cells are inhibited are still poorly elucidated. In some cases, compounds were found to induce cell death by activation of the apoptotic process; nevertheless the mechanisms underlying the apoptosis still need more investigations. Some compounds were found to create an imbalance in cellular redox potential, with mitochondria representing a central role in the process. However, more studies are needed in order to clarify if mitochondria and oxidative stress are the direct targets, or if they are just a consequence of upstream damage. Cell cycle is another disturbed process, mainly due to disruption of the microtubules and actin filaments; however there are only a few studies connecting marine cyanobacterial compounds with alterations in cell cycle and more studies are needed in order to clarify the involvement of these compounds in the process. Not surprisingly, the proteins directly involved in apoptosis, caspases, non-caspases proteases and the Bcl-2 protein family, also seem to be associated with the cyanobacterial compounds activity. Even membrane sodium channels can establish interactions with the compounds, revealing its potentially important role in the observed effects.

In summary, marine cyanobacteria seems to be clearly an important source of anticancer drugs. However, more investigations are needed in order to clarify the specific targets and the mechanisms that are behind cancer cell cytotoxicity, namely the involvement of the apoptotic process. 
